# Magnetic Resonance Imaging in (Near-)Term Infants with Hypoxic-Ischemic Encephalopathy

**DOI:** 10.3390/diagnostics12030645

**Published:** 2022-03-06

**Authors:** Corline E. J. Parmentier, Linda S. de Vries, Floris Groenendaal

**Affiliations:** 1 Department of Neonatology, University Medical Center Utrecht, 3584 EA Utrecht, The Netherlands; e.j.parmentier-2@umcutrecht.nl (C.E.J.P.); l.s.devries@umcutrecht.nl (L.S.d.V.); 2 Department of Neonatology, Leiden University Medical Center, 2333 ZA Leiden, The Netherlands

**Keywords:** perinatal asphyxia, hypoxic-ischemic encephalopathy, neonatal encephalopathy, neonatal neuroimaging, magnetic resonance imaging, diffusion-weighted imaging, magnetic resonance spectroscopy, outcome prediction

## Abstract

Hypoxic-ischemic encephalopathy (HIE) is a major cause of neurological sequelae in (near-)term newborns. Despite the use of therapeutic hypothermia, a significant number of newborns still experience impaired neurodevelopment. Neuroimaging is the standard of care in infants with HIE to determine the timing and nature of the injury, guide further treatment decisions, and predict neurodevelopmental outcomes. Cranial ultrasonography is a helpful noninvasive tool to assess the brain before initiation of hypothermia to look for abnormalities suggestive of HIE mimics or antenatal onset of injury. Magnetic resonance imaging (MRI) which includes diffusion-weighted imaging has, however, become the gold standard to assess brain injury in infants with HIE, and has an excellent prognostic utility. Magnetic resonance spectroscopy provides complementary metabolic information and has also been shown to be a reliable prognostic biomarker. Advanced imaging modalities, including diffusion tensor imaging and arterial spin labeling, are increasingly being used to gain further information about the etiology and prognosis of brain injury. Over the past decades, tremendous progress has been made in the field of neonatal neuroimaging. In this review, the main brain injury patterns of infants with HIE, the application of conventional and advanced MRI techniques in these newborns, and HIE mimics, will be described.

## 1. Introduction

Perinatal asphyxia is a major cause of morbidity and mortality in term infants worldwide. It refers to the interruption of blood flow or impaired gas exchange around labor and delivery, which can result in multi-organ failure [1]. The most important consequence of perinatal asphyxia is brain involvement, known as hypoxic-ischemic encephalopathy (HIE). The incidence of HIE is estimated at 1–2 per 1000 live births in high-income countries and rises up to approximately 4–40 per 1000 live births in developing countries [2,3]. Therapeutic hypothermia started within 6 h after birth is currently the only neuroprotective treatment that has been proven to reduce death and neurological sequelae in term infants with moderate to severe HIE [4]. Nevertheless, many infants still experience adverse outcomes despite cooling, including cerebral palsy (CP), cognitive, visual or hearing impairment, and even death [5]. Moreover, a significant proportion of infants with mild HIE, who are currently not eligible for therapeutic hypothermia, have an adverse outcome at follow-up [6].

Neuroimaging is widely used to aid in the diagnosis and neurodevelopmental prognostication in infants with HIE. Although HIE is responsible for the majority of newborns with encephalopathy, other etiologies, including infectious diseases, metabolic disorders, trauma and congenital disorders, can mimic HIE and should also be considered in the diagnostic work-up [7,8]. In addition to the clinical history, physical and neurological examination, and neuromonitoring, the pattern and extent of brain injury on neuroimaging can elucidate the underlying etiology and timing of brain injury. Furthermore, quantification of the degree of brain injury is important to guide decisions on the redirection of care or application of future additional neuroprotective interventions and to predict neurodevelopmental outcomes.

Multiple neuroimaging techniques are available to visualize brain injury in the neonatal period. Cranial ultrasonography is a helpful noninvasive tool to detect antenatal onset of injury and abnormalities that may mimic HIE. Abnormalities related to HIE can be recognized with ultrasonography as well, but it will take 48–72 h to see these changes develop in the white matter or deep gray nuclei [9]. Magnetic resonance imaging (MRI) which includes diffusion-weighted imaging (DWI) is the preferred imaging modality during the first week after birth to determine the extent of brain injury and predict neurodevelopmental outcome in infants with symptoms of HIE, without its prognostic utility being altered by therapeutic hypothermia [9,10,11,12,13]. In addition, proton magnetic resonance spectroscopy (^1^H-MRS) is increasingly being used to assess metabolic changes and improve prognostication [14,15]. Advanced MRI techniques, including diffusion tensor imaging (DTI) and arterial spin labeling (ASL), can provide additional information on white matter microstructure and cerebral perfusion, and further improve prognostication [16,17,18,19]. In this review, the different patterns of brain injury in term infants with HIE, the neuroimaging characteristics of HIE mimics, and the application of conventional and advanced MRI techniques to detect brain injury and predict the neurodevelopmental outcome of infants with perinatal asphyxia will be addressed.

## 2. Brain Injury Patterns in HIE

The extent and location of brain injury in infants with perinatal asphyxia is dependent on the severity, timing and duration of the hypoxic-ischemic event, in addition to the brain maturation at the time the insult occurs [20]. Based on animal and human studies, three main patterns of injury can be distinguished in infants with HIE:

### 2.1. Basal Ganglia and Thalami (BGT) Predominant Pattern of Injury

**Figure 1:**
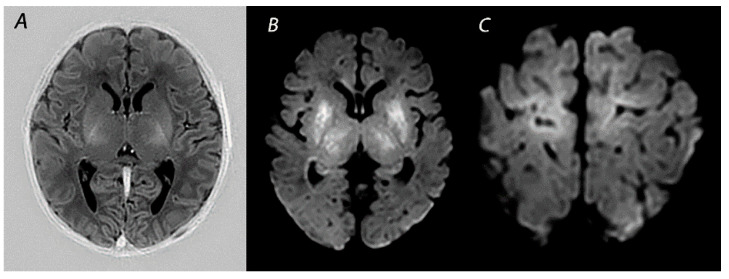
MRI performed on day 5 in a newborn born with Apgar scores 0/2/5 who received therapeutic hypothermia for HIE. Although the signal intensity of the posterior limb of the internal capsule appears normal on T1-weighted imaging (**A**), DWI shows extensive diffusion restriction in the basal ganglia and thalami (**B**), and the perirolandic cortex (**C**). The infant died after redirection of care.

Multiple conditions can lead to an abrupt impairment of placental perfusion or oxygen delivery in the umbilical cord, including placental abruption, umbilical cord prolapse, shoulder dystocia and uterine rupture, resulting in acute profound asphyxia. As there is insufficient time to redirect blood flow through autoregulation, these sentinel events will especially affect the brain areas with the highest metabolic demand. These include the basal ganglia and thalami (BGT), mainly the ventrolateral thalami and posterior putamina, and the perirolandic cortex (Figure 1) [21]. These lesions are associated with impaired motor neurodevelopment, including CP [22,23]. Martinez-Biarge and colleagues demonstrated a strong association between the severity of BGT abnormalities and the severity of motor impairment, with a predictive accuracy of severe BGT lesions being 0.89 (95% CI 0.83–0.96) for severe motor impairment [22].

### 2.2. White Matter/Watershed (WM/WS) Predominant Pattern of Injury

**Figure 2:**
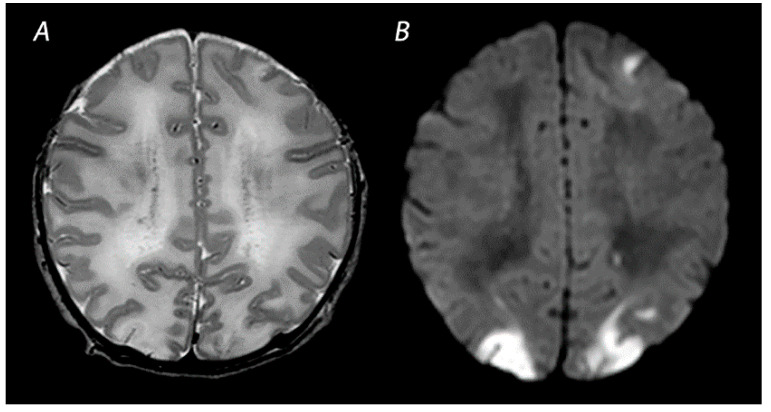
MRI was performed on day 4 in a term infant who was born with Apgar scores of 1/5. Amplitude integrated EEG initially showed a continuous normal voltage background pattern and cooling was not started. The infant developed seizures at 10 h after birth. MRI shows a watershed predominant pattern of injury, with T2-weighted imaging showing loss of distinction between the gray and white matter at the cortex of the occipital lobes and the left frontal lobe (**A**) and DWI (**B**) showing diffusion restriction in these areas. The infant had a normal outcome at 18 months of age.

Mild to moderate asphyxia, also referred to as partial prolonged asphyxia, allows time for cerebral autoregulation to redirect blood flow to the high metabolic brain areas, at the cost of the vascular watershed areas of the anterior, middle and posterior cerebral arteries [20]. This results in a watershed predominant pattern of injury (Figure 2). As injury follows a parasagittal distribution along these vascular border zones, this pattern is also known as the “parasagittal pattern of injury” [24]. In the more severely affected infants, injury can also be seen in the parasagittal cortex and the subcortical white matter. Physiological and neurological manifestations in infants with this type of injury are often not severe and only present for a short time after birth, in contrast to infants with BGT pattern of injury who in general, have lower Apgar scores, need more intensive resuscitation at birth, and present with more severe encephalopathy [25]. Nevertheless, clinical and neurological abnormalities may progress over time, highlighting the importance of careful observation of infants with perinatal asphyxia [26]. Severe motor impairment is rare in infants with mild to moderate WM/WS predominant patterns of injury [25,27]. At early follow-up, 12–18 months after birth, they are often considered to have a normal outcome [28]. These infants are, however, likely to grow into their deficits and develop cognitive problems over the years, highlighting the importance of long-term follow-up to detect disabilities early in life and ensure an early start of interventions [25,27,29,30].

Some infants with HIE show abnormalities restricted to the periventricular white matter, including punctate white matter lesions (PWMLs), small focal patches of increased signal intensity on T1-weighted imaging, and decreased intensity on T2-weighted imaging [31,32]. Hayman et al. studied 44 (near-)term infants with PWMLs, of whom 45% were associated with perinatal asphyxia [31]. None of these infants had evidence of BGT injury or another more extensive pattern of injury. The majority of infants in this study had normal early outcomes or mild neurologic deficits. Most of those who experienced severe neurological sequelae had an underlying genetic disorder.

### 2.3. Near Total Injury

**Figure 3:**
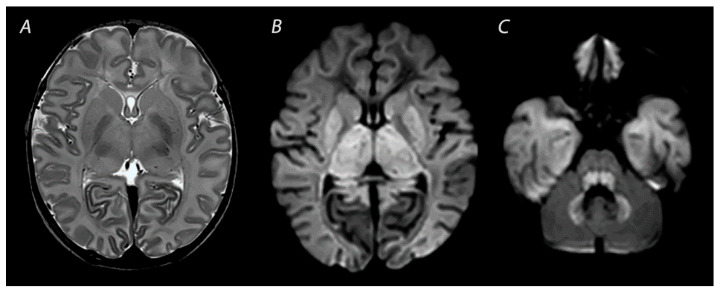
MRI performed on day 3 after birth, showing near total injury with extensive injury in the white matter, BGT, brainstem and cerebellum. Abnormalities are often more subtle on T2 weighted imaging in the first days after the hypoxic-ischemic insult (**A**) and are best seen using DWI on early MRI (**B**,**C**). Except for the dentate nuclei, the signal in the cerebellum is not increased and therefore the term ‘the white brain’ was coined. The infant died after redirection of care.

Near total injury, which is also referred to as “global injury”, is characterized by diffuse injury in both the BGT and white matter. This pattern is seen in infants affected by very severe and prolonged asphyxia and are therefore less often reported compared to BGT or WM/WS patterns of injury, as these infants may be too ill to be scanned after birth and pass away before MRI can be performed [25] (Figure 3). The cerebellum may look relatively normal on DWI in contrast to a completely white cerebrum, which is the reason why this pattern is also being referred to as the “white cerebrum” [33]. Nevertheless, it has been demonstrated that cerebellar abnormalities may be underestimated on DWI, and that only in cases of very severe cytotoxic edema abnormalities are found [34]. There is growing evidence for a superior role of advanced MRI modalities, such as quantitative DTI analysis, to adequately detect cerebellar injury [35].

### 2.4. Other Injury Associated with HIE

**Figure 4:**
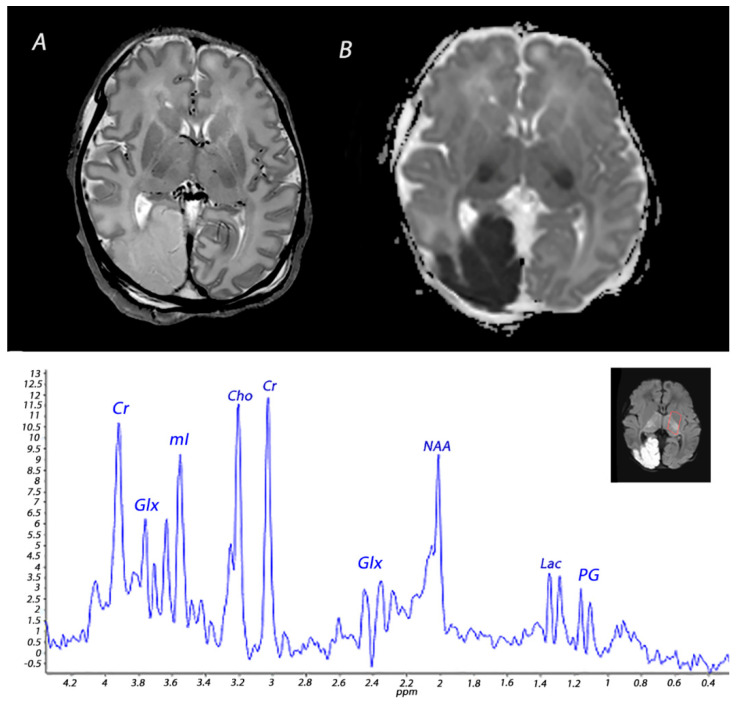
MRI performed on day 3 in an infant born with Apgar scores of 3/4/6 who received therapeutic hypothermia for severe HIE. MRI showed posterior cerebral artery stroke, presenting as increased signal intensity and loss of cortical gray and white matter differentiation on T2 weighted imaging (**A**) and a low signal intensity on ADC mapping (**B**). Injury was also noted in the BGT. ^1^H-MRS performed in the BGT shows a lactate peak, presenting as a doublet peak at 1.33 ppm.

Vascular abnormalities, including perinatal arterial ischemic stroke, sinovenous thrombosis and perinatal hemorrhagic stroke, have also been demonstrated in infants with encephalopathy due to perinatal asphyxia [36,37]. Previous studies have demonstrated perinatal stroke in 4–5% of newborns with HIE [37,38] (Figure 4). Whether there is a causal relationship between perinatal asphyxia and neonatal stroke is controversial. In experimental and clinical studies, asphyxia has been proposed to be involved in the development of neonatal stroke [39,40]. Findings from a study by Harbert and colleagues have suggested that therapeutic hypothermia could potentially reduce seizures after perinatal stroke [41]. The exact etiology of this disease, however, remains to be further elucidated.

**Figure 5:**
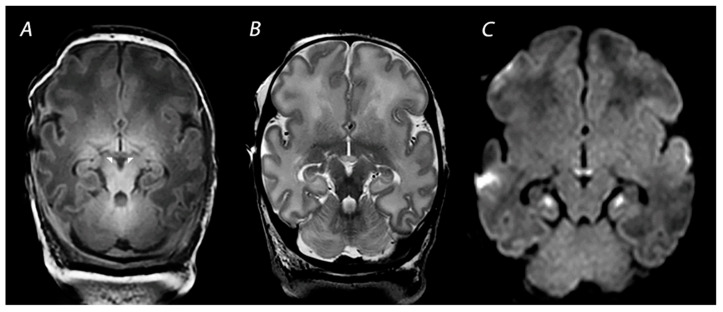
MRI performed on 10 days after birth showing injury of the mammillary bodies, presenting as decreased signal intensity on T1 weighted imaging (indicated by the white arrows on (**A**), swelling and increased signal intensity on T2-weighted imaging (**B**), and diffusion restriction on diffusion-weighted imaging (**C**). Diffusion restriction is also noted in the hippocampi, which is often seen in combination with mammillary body injury.

Some infants with HIE demonstrate brain injury in the limbic system, particularly in the hippocampal region, which can lead to long-term episodic memory impairment at school age [42,43]. Additionally, a recent study has demonstrated that the mammillary bodies (MB), which are also part of the limbic circuit and play an important role in episodic memory, are often injured in infants with HIE [44] (Figure 5). Molavi and colleagues showed that 13% of full-term infants with HIE had abnormal signal intensity on T2-weighted imaging. In all but one of the infants with follow-up MRI, these initial signal abnormalities resulted in MB atrophy [44]. In another study across multiple centers, it was demonstrated that approximately 40% of infants with HIE who had received hypothermia had signal abnormalities in the MBs [45]. Annink et al. reported that MB atrophy was significantly associated with lower intelligence quotients, verbal long-term memory scores, and visual-spatial long-term memory scores at 10 years of age [46].

## 3. Magnetic Resonance Imaging in Infants with HIE

### 3.1. Conventional MRI

MRI has been performed in newborns with HIE since the late 1980s, and initially included T1- and T2-weighted imaging [47]. The introduction of DWI in the mid-1990s has greatly improved the assessment of brain lesions in newborns with asphyxia. T1-weighted imaging aids in the assessment of myelination, the depiction of ischemia, and the detection of subacute hemorrhage in regions with important prognostic implications, such as the BGT and the brainstem [48]. T2-weighted sequences are useful to identify abnormalities in the white matter and to assess the gray-white matter differentiation at the cortex [48]. Abnormalities in this delineation can reveal cortical injury, also referred to as “loss of the cortical ribbon” (Figure 2). During the first days after birth, the abnormalities on T1- and T2-weighted imaging are often subtle, but they become gradually more apparent by the second half of the first week [49]. It has been demonstrated that the absence of normal high-signal intensity of the posterior limb of the internal capsule (PLIC) on T1- and T2-weighted imaging is highly predictive of severe adverse motor outcomes [22,50]. However, these results derived from studies performing MRI in both the first and second week after birth, and one should be aware that an abnormal signal of the PLIC may take up to 4 days to become visible when only T1- and T2-weighted imaging are used (Figure 1) [22].

### 3.2. Diffusion Weighted Imaging

DWI is an imaging modality based upon the measurement of the random motion of water molecules within a voxel of brain tissue, providing information on the functional state of the brain tissue. The apparent diffusion coefficient (ADC) is an indicator of the magnitude of movement of water protons within the tissue. As the ADC indicates the overall diffusion restriction, low ADC values will cause a high-signal intensity on isotropic DWI [51]. A variety of studies have demonstrated restricted water diffusion due to hypoxic-ischemic injury, which can be visible at an early stage when conventional T1 and T2 sequences are still inconspicuous [52,53]. In general, abnormalities seen on DWI peak at 3–5 days after the insult and subsequently normalize at approximately 11–12 days for infants treated with therapeutic hypothermia and 6–8 days in non-cooled infants. One must keep in mind that DWI obtained within the first day after birth and near the time of pseudo-normalization may lead to an underestimation of the extent of brain injury [54].

### 3.3. Susceptibility Weighted Imaging

**Figure 6:**
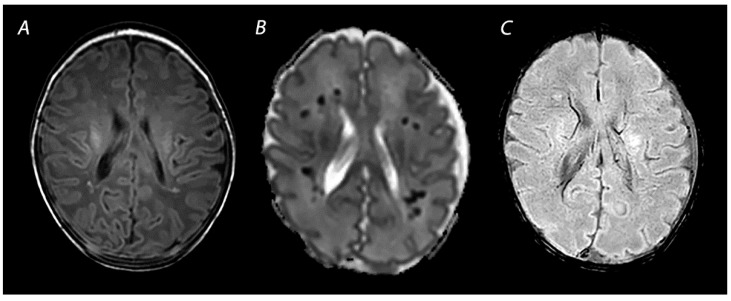
MRI performed on day 7 showing punctate white matter lesions. While these lesions are subtle on T1 weighted imaging (**A**), the abnormalities are much clearer and more numerous on the ADC map (**B**). Susceptibility weighted imaging can be used to rule out hemorrhagic abnormalities (**C**).

Susceptibility weighted imaging (SWI) is a three-dimensional MRI modality with high spatial resolution and flow compensation that accentuates the magnetic properties of iron, blood, blood products and calcifications [55]. It is therefore exquisitely useful to detect vascular abnormalities, such as hemorrhages and cerebral vascular malformations (Figure 6). As this sequence is very sensitive to the detection of blood oxygenation changes within the veins, due to the increased magnetic susceptibility of deoxygenated blood, SWI can also play an important role in the early detection of HIE and prognostication [56,57]. The compensatory increase in the oxygen extraction fraction following hypoxia-ischemia results in an increased level of deoxygenated hemoglobin in the cerebral vessels draining the injured areas. This may be depicted in SWI as prominent hypo-intense veins. SWI may reveal vascular brain abnormalities even before cytotoxic edema is visible on DWI [58].

## 4. Advanced Imaging Modalities

### 4.1. Diffusion Tensor Imaging

In contrast to DWI, DTI also accounts for the effects of the diffusion directionality of water, which is expressed as functional anisotropy (FA). DTI is a powerful tool to assess microstructural abnormalities of the brain. However, while abnormalities on DWI can be interpreted visually, the acquisition and analysis of DTI data are significantly more complex and, in general, require a reliable quantitative analysis method to draw meaningful conclusions [59]. One of these methods is the region of interest analysis, in which diffusion measures are obtained from a specific brain region, defined by manual delineation or automated parcellation or segmentation. In a systematic review including 19 studies on DTI, Dibble and colleagues identified three key regions of interest showing altered diffusion in newborns with HIE: the PLIC, and the genu and splenium of the corpus callosum [14]. Lower FA values in these areas were associated with worse neurodevelopmental outcomes. Another method for quantitative analysis is tract-based spatial statistics (TBSS). TBSS is an automated objective observer-independent whole-brain approach that analyzes aligned FA images from multi-subject diffusion data, allowing powerful comparisons of DTI without requiring a subjective prespecification of regions of interest [59]. Using TBSS, Porter and colleagues demonstrated widespread microstructural abnormalities in white matter tracts in full-term infants with HIE, which were reduced after therapeutic hypothermia, showing that TBSS is a suitable method to compare white matter tissue integrity among infants and a promising biomarker for early phase trials of new neuroprotective strategies [60]. In another study including 43 term infants with HIE, Tusor et al. demonstrated that DTI analyzed by TBSS could predict 2-year outcomes of these newborns, with those having an adverse outcome showing significantly lower FA values in the corpus callosum, the centrum semiovale, anterior and posterior limb of the internal capsule, cerebral peduncles, optic radiation, external capsule, fornix, cingulum and inferior longitudinal fasciculus [18].

### 4.2. Arterial Spin Labeling

ASL is a noninvasive technique to measure brain perfusion, using magnetically labeled water protons as an endogenous tracer [61]. Following the acute phase of injury after the hypoxic-ischemic event, impaired autoregulation often results in cerebral hyperperfusion, which can lead to delayed brain injury and correlates with an adverse neurodevelopmental outcome [62].

Perfusion abnormalities visualized with ASL have been shown to aid in early diagnosis and prognostication of infants with HIE [16,63,64]. Wintermark and colleagues assessed brain perfusion using ASL in the first week after birth in 18 term infants with perinatal asphyxia, of whom 11 were treated with TH, and 4 were control patients and demonstrated that abnormal cerebral blood flow values can precede injury seen on later MRI [63]. Cooled infants who developed evidence of hypoxic-ischemic brain injury on MRI showed more markedly decreased perfusion on the first day after birth in the brain areas subsequently exhibiting injury, in comparison to the control group and the cooled infants who did not develop brain injury. On the second day after birth, all but one of these infants demonstrated hyperperfusion in these injured brain areas, suggesting that an initial hypoperfusion phase followed by a hyperperfusion phase in specific brain regions correlates with injury development in those regions. Asphyxiated neonates who did not receive therapeutic hypothermia and developed brain injury displayed hyperperfusion on days 1–6 after birth in the brain areas showing injury on MRI. Another study evaluated the predictive value of ASL in 28 infants with HIE and reported that perfusion in the BGT was higher in those having an adverse outcome at 9–18 months of age than in the infants with a favorable outcome [16]. When comparing the predictive value of ASL with other MRI predictive markers, the combination of ASL with Lactate/N-acetylaspartate (NAA) measured by ^1^H-MRS, was demonstrated to be the best predictor of outcome. ASL seems most relevant when performed in the first days after the hypoxic-ischemic insult. Based on a study on newborns with HIE who underwent MRI around 4 days and 11 days after birth, Proisy and colleagues demonstrated that cerebral blood flow levels measured in the BGT around day 4 were significantly higher in infants with ischemic lesions on MRI compared to infants with normal MRI [65]. No significant difference in cerebral blood flow levels measured in the BGT around 11 days after birth was demonstrated between these groups. However, this could also be due to the small sample size of this study, with only 28 infants having MRI around 4 days after birth, and 23 around 11 days after birth.

## 5. Magnetic Resonance Spectroscopy

Nuclear magnetic resonance spectroscopy of protons, referred to as ^1^H-MRS, has been used since the early 1990s to obtain metabolic information about brain metabolism and detect changes in neurochemistry caused by perinatal asphyxia [66]. The magnetic field that a particular proton experiences is influenced by the chemical environment to which it is subjected. Differently sited protons will therefore resonate at different frequencies. This so-called chemical shift is used in ^1^H-MRS to discern different metabolites. There is compelling evidence for the excellent prognostic value of ^1^H-MRS in infants with HIE [13,14]. NAA and lactate measured in the deep gray matter have been demonstrated to be the most valuable biomarkers in the assessment of metabolic changes associated with HIE [15]. The predictive value for an adverse outcome is excellent, and not affected by the use of therapeutic hypothermia [13,15]. These metabolites have a long T2 relaxation time and are therefore best detected using a long (>130 ms) echo-time (TE), which improves the signal-to-noise ratio of these metabolites due to the attenuation of signals from metabolites with a shorter time of relaxation.

NAA is found in high concentrations in neurons and has been described as a surrogate biomarker of neuronal integrity and function [67]. Total NAA (tNAA; NAA + N-acetylaspartylglutamate) in the deep gray matter was shown to be significantly lower in infants with adverse outcomes at 18–22 months of age during the first 2 weeks after birth [68]. In a prospective multicenter cohort study, Lally et al. demonstrated that using ^1^H-MRS, thalamic NAA concentration alone could accurately predict an adverse neurodevelopmental at 18–24 months of age, with a sensitivity of 100% and specificity of 97% [14]. However, measuring absolute concentrations of metabolites is difficult, as the detection level of NAA falls in the millimolar range and requires internal or external standards. Although water-unsuppressed concentration can be used as an internal standard, this may be unreliable as the amount of intracellular and extracellular brain water may be affected by perinatal asphyxia [69]. Metabolite ratios such as NAA/choline are often used instead and have also been demonstrated to be predictive of a poor outcome in newborns with HIE [70].

Lactate is a product of anaerobic metabolism, hence increased in hypoxic-ischemic conditions. Lactate has a chemical shift of 1.33 ppm and presents as a doublet peak on spectra obtained with a short TE (35–40 ms) or long TE (272–288 ms), and an inverted peak with a TE around 136–144 ms (Figure 4). Changes in lactate concentrations due to HIE are often transient; a study analyzing cerebral lactate concentrations in 52 infants with HIE undergoing hypothermia showed high levels of lactate during hypothermia in infants with moderate-severe HIE, which slightly decreased after cooling and subsequently normalized by the end of the first week after birth [71]. The cerebral lactate remained low in infants with no or mild brain injury during the first 2 weeks after birth. These temporal resolutions are important to take into account when interpreting ^1^H-MRS findings. In a recent paper, it was demonstrated that during the first 24–96 h after birth tNAA concentration, ADC values, lactate levels, and lactate/tNAA ratios all had high prognostic value in infants with HIE undergoing therapeutic hypothermia, but only tNAA retained its good prognostic value in the second week after birth [72]. Furthermore, it is important to consider gestational age when interpreting ^1^H-MRS spectra. The presence of a lactate peak may be normal in preterm infants, and they may show relatively decreased NAA levels compared to term infants [73,74].

In the past, phosphorus MRS (^31^P-MRS) was used to study brain energy metabolism in human infants. Although an association between ^31^P-MRS findings and neurodevelopmental outcomes has been demonstrated in infants with birth asphyxia, it is not routinely performed in the clinical setting [75,76].

## 6. HIE Mimics

Although HIE is the most important underlying etiology of neonatal encephalopathy, a variety of other conditions can present with similar symptoms [7]. The differential diagnosis of neonatal encephalopathy includes vascular abnormalities, metabolic disorders, central nervous system infections, congenital malformations and trauma. The pattern and extent of brain injury on MRI can aid in the investigation of the underlying condition causing encephalopathy.

Although individually rare, inborn errors of metabolism account for a significant number of disorders in the neonatal period and require early diagnosis and therapy to prevent severe neurological sequelae. Among the most commonly found metabolic disorders presenting in the neonatal period are urea cycle defects, organic acidemias and disorders of amino acid metabolism [77]. Metabolic and genetic laboratory testing is required to confirm the diagnosis, but MRI and ^1^H-MRS using a short TE (30–40 ms) may allow targeted investigations by suggesting a certain diagnosis. For example, encephalopathy caused by urea cycle defects may be distinguished from HIE by conventional MRI showing diffuse generalized edema involving the cerebral cortex and the subcortical white matter with an abnormal signal intensity of the globus pallidus and putamen, while sparing the thalamus [77]. A spectroscopic sequence using a short TE enables the identification of the metabolite peaks that can suggest, among others, maple syrup urine disease (methyl peak of branched-chain amino acids at 0.9 ppm) or nonketotic hyperglycinemia (glycine, at 3.55 ppm) [78,79].

**Figure 7:**
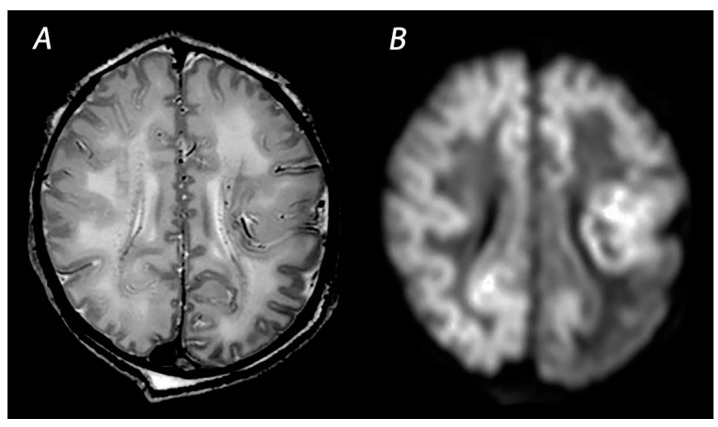
MRI was performed on day 3 in an infant born with Apgar scores of 9/10 who developed intractable seizures within the first hours after birth. T2 weighted imaging shows the decreased distinction of the gray and white matter at the cortex (**A**). DWI shows extensive diffusion restriction with sparing of the anterior periventricular white matter and an asymmetrical distribution of white matter injury in the parieto-occipital lobes (**B**). The infant died on day 5 after redirection of care and was subsequently diagnosed with Molybdenum cofactor deficiency.

An important metabolic disorder that is often confused with HIE is Molybdenum cofactor deficiency (MoCD). MoCD is a rare autosomal recessively inherited disorder that presents during the first days after birth with intractable seizures, feeding difficulties, and progressive neurologic deterioration [80]. Microcephaly and dysmorphic facial features are often present. MRI features include, among others, global cerebral edema, diffuse brain atrophy, arrested development of myelination, gliosis and subsequent cystic necrosis of the white matter [81]. Early MRI may be helpful to distinguish MoCD from HIE. In comparison to HIE, in which deep gray matter injury can range from selective symmetrical involvement of the posterolateral putamina and ventrolateral thalami to more diffuse involvement of the basal ganglia, infants with MoCD have been demonstrated to show more selective subthalamic and pallidal changes in comparison to the rest of the basal ganglia. Other signs indicative of MoCD are pontocerebellar hypoplasia, and the presence of a distinctive cortical/subcortical band of intermediate signal intensity on T2-weighted imaging, outlined by edema on either side, which possibly represents relatively spared parenchyma [80]. When presenting in the immediate neonatal period, extensive diffusion restriction is seen, sometimes with sparing of the cortex (Figure 7). The presence of a lipid peak on ^1^H-MRS, due to white matter destruction, in addition to a reduction in NAA and accumulation of lactate, is also suggestive of MoCD [81]. However, an increase in lipid metabolites is also often seen following hypoxic-ischemic injury [82].

## 7. Scoring Systems

Several MRI scoring systems have been developed to quantify brain injury and predict the outcome of infants with HIE. The first score was described by Barkovich and colleagues, who evaluated MRI by separately assessing injury in the BGT and the watershed regions [83]. The assessment system developed by the National Institute of Child Health and Human Development (NICHD) Neonatal Research Network has developed an approach that grades brain injury using 6 categories (score 0: normal; 1A: lesions in frontal and parietal subcortical areas; 1B: more extensive cerebral lesions in frontal, parietal and occipital subcortical areas; 2A: lesions in BGT and internal capsule; 2B: lesions in BGT, internal capsule and cerebral areas; 3: cerebral hemispheric devastation) [84]. The Rutherford scoring system scores the BGT, PLIC, white matter and cortex. All of these scoring systems were initially based on T1- and T2-weighted imaging.

Recently, Trivedi and colleagues developed a scoring system using serial T1- and T2-weighted sequences and DWI. They graded injury as none, mild, moderate, or severe by assessing abnormalities in six regions: subcortical (BGT and PLIC), white matter, cortex, cerebellum and brainstem [85]. This score was demonstrated to predict an abnormal outcome at 18–24 months after birth with an AUC of 0.72 (95% CI 0.57–0.86).

Weeke et al. developed a comprehensive scoring system combining conventional MRI, including DWI, with ^1^H-MRS [86]. Brain injury is separately categorized into three subgroups: gray matter (BGT, PLIC, perirolandic cortex, brainstem, hippocampus), white matter/cortex (cortex, periventricular white matter, optic radiations, corpus callosum), and cerebellum. Additional findings, including intraventricular and subdural hemorrhage, are also scored. A recent study comparing the Barkovich, NICHD and Weeke scoring systems in relation to the neurodevelopmental outcome at 2 years of age, demonstrated that each of these approaches had good predictive value for cognitive and motor outcome in infants with HIE, but only the Weeke score was associated with language scores at 2 years [87].

## 8. Pitfalls and Recommendations for the Assessment of Brain Injury on MRI

### 8.1. Optimal Timing of MRI

The timing of MRI is of great importance when interpreting the neuroimaging findings, as the patterns of injury on different MRI modalities vary greatly during the first 2 weeks following injury [88]. A summary of the neuroimaging findings in infants with HIE on different MRI modalities is presented in Table 1. The optimal timing of neuroimaging is controversial and differs across centers. Brain injury may continue to evolve over the first days after the hypoxic-ischemic event and is not yet visible at its full extent on T1- and T2-weighted imaging during the first days following birth [49]. Early MRI (i.e., approximately 4–5 days after birth) may however be preferable over late scanning (>7 days after birth) as DWI abnormalities can be assessed before pseudo-normalization has occurred, and MRI can be performed between completion of TH and transfer to another center. Previous studies that compared early to late MRI have demonstrated good agreement between these scans [89,90,91]. Moreover, diffusion and ^1^H-MRS abnormalities have been reported to normalize from 4 to 5 days onwards following the insult and could therefore be missed when MRI is performed in the second week after birth [88]. In a recent paper, O’Kane and colleagues demonstrated a substantial correlation between early (<7 days) and late (≥7 days of age) MRI. However, early MRI was more consistently predictive of abnormal outcomes and more likely to identify severe injury in cases of discrepancy compared with late MRI [91].

Some centers have reported that MRI can be performed during therapeutic hypothermia without affecting the safety and efficacy of this neuroprotective intervention [94,95]. High suspicion of antenatal injuries or cerebral hemorrhage has been proposed as an important clinical indication to perform MRI already during hypothermia, as this would allow adjustment or discontinuation of cooling therapy. However, MRI including DWI performed within 24 h after the hypoxic-ischemic event may be false negative, and cranial ultrasonography remains the first-choice neuroimaging modality to screen for the presence of antenatal injury and cerebral hemorrhage, as it is noninvasive, rapidly available and well-capable of detecting these lesions when performed by trained professionals [96]. Computed tomography should be reserved for emergency situations when cranial ultrasound is not readily available, as ionizing radiation can adversely affect intellectual development and increase the risk for radiation-related malignancies later in life [97,98].

### 8.2. Repeat MRI

In the summary of neuroimaging in infants with HIE published by the executive task force on neonatal HIE from the ACOG in 2014, it was recommended to perform two MRI scans in infants with HIE when feasible. Conventional MRI, including DWI and ^1^H-MRS, should be performed at 1–4 days after birth to assess the timing of injury, and a repeat MRI at 7–21 days after birth was recommended to determine the extent of brain injury [99]. In light of emerging evidence that a single MRI performed in the first week after birth is sufficient to determine the extent of brain injury and predict outcome in newborns with HIE, the Newborn Brain Society Guidelines and Publications Committee recently published updated recommendations for neuroimaging of newborns with HIE, proposing to perform MRI at 2–5 days after birth, typically immediately after cooling, to confirm the diagnosis and predict outcome [100]. A repeat MRI at 10–14 days after birth should only be considered when there is a discrepancy between early MRI and the clinical condition of the infant, or if the first scan has led to ambiguity.

Although rare, MRI beyond the first month after birth is sometimes performed to assess myelination and the evolution of lesions that were seen on the neonatal scan, or when infants were too ill to be scanned within the first weeks after birth [101]. There are studies that have suggested that MRI performed at 2–5 months of age has a predictive significance for later neurodevelopmental outcomes, but literature on MRI at this age is scarce [101,102,103].

## 9. Conclusions

Cerebral MRI including DWI, is the gold standard for the assessment of brain injury in infants with HIE. The pattern and extent of injury indicate the timing and severity of the hypoxic-ischemic insult and provide important information to predict neurodevelopmental outcomes. Complementary information about the metabolic changes can be provided by ^1^H-MRS, which has been proven to be the most reliable biomarker in the prognostication of newborns with HIE. Over the past decades, tremendous progress has been made to refine the evaluation of brain injury in infants with HIE, and advanced imaging modalities are increasingly being used to maximize the information obtained from neonatal MRI. The timing of MRI is of great importance in the interpretation of imaging findings, as patterns of injury vary significantly over the first weeks after birth, dependent on the imaging modality used. MRI including DWI and ^1^H-MRS performed immediately following therapeutic hypothermia (i.e., 4–5 days after birth) is optimal for the diagnosis and prognostication of infants with HIE, as diffusion abnormalities peak at 3–5 days after birth, and pseudo-normalization of DWI and ^1^H-MRS occurs by the end of the first week. Different scoring systems have been developed to quantify brain injury on neonatal MRI and have been demonstrated to be well-capable of predicting neurodevelopmental outcomes of infants with HIE. However, although perinatal asphyxia is by far the most common cause of encephalopathy, it is important to be aware of causes that can mimic HIE, and MRI should always be interpreted in the context of the clinical history, findings from the physical and neurological examination, and information obtained from laboratory testing, neuromonitoring, and other neuroimaging modalities (e.g., cranial ultrasonography) in newborns presenting with encephalopathy.

## Figures and Tables

**Table 1:** Imaging findings and time frame for assessment of abnormalities for each MRI modality.

Pattern of Injury	Imaging Modality	Imaging Findings	Time Frame of Abnormalities
WM/WS	T1WI/T2WI	Abnormal signal intensity in the white matter of the watershed areas of the cerebral arteries, and also the overlying cortex in severely affected infants. T2WI may show loss of gray-white matter differentiation at the cortex.	Inconspicuous or subtle abnormalities in the first days, which become gradually more apparent by the latter half of the first week following the insult. MRI obtained beyond 1 month can show cortical thinning, white matter volume loss, cysts and gliosis of the cortex and white matter.
	DWI	High signal intensity on isotropic DWI with low ADC values in the affected areas.	Abnormalities peak at 3–5 days after the insult. Pseudo-normalization occurs after approximately 11–12 days for infants treated with therapeutic hypothermia, and 6–8 days in non-cooled infants.
	^1^H-MRS	Increased lactate and decreased NAA in the affected white matter.	Lactate in general increases <24 h and subsequently normalizes by the end of the first week, but persistent elevation has been reported. ^1^ NAA declines <24 h and remains low during the first 2 weeks after the insult, although some studies have reported that NAA levels do not significantly diminish until approximately 48 h after the insult. ^2^
	SWI	Prominent hypo-intense veins, low signal intensity at the site of hemorrhagic lesions.	Prominent hypo-intense veins have been observed as early as 18 h after birth, but current literature is limited. Low signal intensity at the site of hemorrhagic lesions is seen immediately and can persists for many months.
BGT	T1WI/T2WI	Abnormal signal intensity in the basal ganglia, thalami and the perirolandic cortex. Absence of a normal high-signal intensity of the PLIC.	Inconspicuous or subtle abnormalities in the first days, which become gradually more apparent by the latter half of the first week following the insult. MRI obtained beyond 1 month can show volume loss, cysts, gliosis and impaired myelination of the central gray matter and perirolandic cortex.
	DWI	High signal intensity on isotropic DWI with low ADC values in affected areas.	Abnormalities peak at 3–5 days after the insult. Pseudo-normalization occurs after approximately 11–12 days for infants treated with therapeutic hypothermia, and 6–8 days in non-cooled infants.
	^1^H-MRS	Increased lactate and decreased NAA in basal ganglia and thalami.	Lactate in general increases <24 h and subsequently normalizes by the end of the first week, but persistent elevation has been reported. ^1^ NAA declines <24 h and remains low during the first 2 weeks after the insult, although some studies have reported that NAA levels do not significantly diminish until approximately 48 h after the insult. ^2^
	SWI	Prominent hypo-intense veins	Prominent hypo-intense veins have been observed as early as 18 h after birth, but current literature is limited.

^1^—Based on previous work by Barkovich et al. [88]; ^2^—Reported by Barkovich et al. [92] and Penrice et al. [93]. ADC: apparent diffusion coefficient; BGT: basal ganglia and thalami; DWI: diffusion-weighted imaging; 1H-MRS: proton magnetic resonance spectroscopy; MRI: magnetic resonance imaging; NAA: N-acetylaspartate; PLIC: posterior limb of the internal capsule; SWI: susceptibility weighted imaging; T1WI: T1-weighted imaging; T2WI: T2-weighted imaging; WM/WS: white matter/watershed.

## Data Availability

Not applicable.

## References

[1] Ahearne C.E., Boylan G.B., Murray D.M. (2016). Short and long term prognosis in perinatal asphyxia: An update. World J. Clin. Pediatr..

[2] Kurinczuk J.J., White-Koning M., Badawi N. (2010). Epidemiology of neonatal encephalopathy and hypoxic-ischaemic encephalopathy. Early Hum. Dev..

[3] Lee A.C., Kozuki N., Blencowe H., Vos T., Bahalim A., Darmstadt G.L., Niermeyer S., Ellis M., Robertson N.J., Cousens S. (2013). Intrapartum-related neonatal encephalopathy incidence and impairment at regional and global levels for 2010 with trends from 1990. Pediatr Res..

[4] Jacobs S.E., Berg M., Hunt R., Tarnow-Mordi W.O., Inder T.E., Davis P.G. (2013). Cooling for newborns with hypoxic ischaemic encephalopathy. Cochrane Database Syst. Rev..

[5] Azzopardi D., Strohm B., Marlow N., Brocklehurst P., Deierl A., Eddama O., Goodwin J., Halliday H.L., Juszczak E., Kapellou O. (2014). Effects of hypothermia for perinatal asphyxia on childhood outcomes. N. Engl. J. Med..

[6] Conway J.M., Walsh B.H., Boylan G.B., Murray D.M. (2018). Mild hypoxic ischaemic encephalopathy and long term neurodevelopmental outcome—A systematic review. Early Hum. Dev..

[7] Karamian A.S., Mercimek-Andrews S., Mohammad K., Molloy E., Chang T., Chau V., Murray D., Wusthoff C.J. (2021). Neonatal encephalopathy: Etiologies other than hypoxic-ischemic encephalopathy. Semin. Fetal Neonatal Med..

[8] Pressler R.M., Cilio M.R., Mizrahi E.M., Moshé S.L., Nunes M.L., Plouin P., Vanhatalo S., Yozawitz E., de Vries L.S., Vinayan K.P. (2021). The ILAE classification of seizures and the epilepsies: Modification for seizures in the neonate. Position paper by the ILAE task force on neonatal seizures. Epilepsia.

[9] Annink K.V., De Vries L.S., Groenendaal F., Vijlbrief D.C., Weeke L.C., Roehr C.C., Lequin M., Reiss I., Govaert P., Benders M.J.N.L. (2020). The development and validation of a cerebral ultrasound scoring system for infants with hypoxic-ischaemic encephalopathy. Pediatr. Res..

[10] Epelman M., Daneman A., Kellenberger C.J., Aziz A., Konen O., Moineddin R., Whyte H., Blaser S. (2010). Neonatal encephalopathy: A prospective comparison of head US and MRI. Pediatr. Radiol..

[11] Cheong J.L., Coleman L., Hunt R.W., Lee K.J., Doyle L.W., Inder T.E., Jacobs S.E., Infant Cooling Evaluation Collaboration (2012). Prognostic utility of magnetic resonance imaging in neonatal hypoxic-ischemic encephalopathy substudy of a randomized trial. Arch. Pediat. Adol. Med..

[12] Rutherford M., Ramenghi L.A., Edwards A.D., Brocklehurst P., Halliday H., Levene M., Strohm B., Thoresen M., Whitelaw A., Azzopardi D. (2010). Assessment of brain tissue injury after moderate hypothermia in neonates with hypoxic-ischaemic encephalopathy: A nested substudy of a randomised controlled trial. Lancet Neurol..

[13] Alderliesten T., de Vries L.S., Benders M.J., Koopman C., Groenendaal F. (2011). MR imaging and outcome of term neonates with perinatal asphyxia: Value of diffusion-weighted MR imaging and (1)H MR spectroscopy. Radiology.

[14] Lally P.J., Montaldo P., Oliveira V., Soe A., Swamy R., Bassett P., Mendoza J., Atreja G., Kariholu U., Pattnayak S. (2019). Magnetic resonance spectroscopy assessment of brain injury after moderate hypothermia in neonatal encephalopathy: A prospective multicentre cohort study. Lancet Neurol..

[15] Thayyil S., Chandrasekaran M., Taylor A., Bainbridge A., Cady E.B., Chong W.K.K., Murad S., Omar R.Z., Robertson N.J. (2010). Cerebral magnetic resonance biomarkers in neonatal encephalopathy: A meta-analysis. Pediatrics.

[16] De Vis J.B., Hendrikse J., Petersen E.T., de Vries L.S., van Bel F., Alderliesten T., Negro S., Groenendaal F., Benders M.J. (2015). Arterial spin-labelling perfusion MRI and outcome in neonates with hypoxic-ischemic encephalopathy. Eur. Radiol..

[17] Dibble M., O’Dea M.I., Hurley T., Byrne A., Colleran G., Molloy E.J., Bokde A.L.W. (2020). Diffusion tensor imaging in neonatal encephalopathy: A systematic review. Arch. Dis. Child Fetal..

[18] Tusor N., Wusthoff C., Smee N., Merchant N., Arichi T., Allsop J.M., Cowan F.M., Azzopardi D., Edwards A.D., Counsell S.J. (2012). Prediction of neurodevelopmental outcome after hypoxic-ischemic encephalopathy treated with hypothermia by diffusion tensor imaging analyzed using tract-based spatial statistics. Pediatr. Res..

[19] Massaro A.N., Evangelou I., Fatemi A., Vezina G., McCarter R., Glass P., Limperopoulos C. (2015). White matter tract integrity and developmental outcome in newborn infants with hypoxic-ischemic encephalopathy treated with hypothermia. Dev. Med. Child Neurol..

[20] Gunn A.J., Bennet L. (2009). Fetal hypoxia insults and patterns of brain injury: Insights from animal models. Clin. Perinatol..

[21] Okereafor A., Allsop J., Counsell S.J., Fitzpatrick J., Azzopardi D., Rutherford M.A., Cowan F.M. (2008). Patterns of brain injury in neonates exposed to perinatal sentinel events. Pediatrics.

[22] Martinez-Biarge M., Diez-Sebastian J., Kapellou O., Gindner D., Allsop J.M., Rutherford M.A., Cowan F.M. (2011). Predicting motor outcome and death in term hypoxic-ischemic encephalopathy. Neurology.

[23] Martinez-Biarge M., Diez-Sebastian J., Rutherford M.A., Cowan F.M. (2010). Outcomes after central grey matter injury in term perinatal hypoxic-ischaemic encephalopathy. Early Hum. Dev..

[24] Volpe J.J., Pasternak J.F. (1977). Parasagittal cerebral injury in neonatal hypoxic-ischemic encephalopathy: Clinical and neuroradiologic features. J. Pediatr..

[25] Miller S., Ramaswamy V., Michelson D., Barkovich A.J., Holshouser B., Wycliffe N., Glidden D., Deming D., Partridge J.C., Wu Y.W. (2005). Patterns of brain injury in term neonatal encephalopathy. J. Pediatr..

[26] Sato Y., Hayakawa M., Iwata O., Okumura A., Kato T., Hayakawa F., Kubota T., Maruyama K., Hasegawa M., Sato M. (2008). Delayed neurological signs following isolated parasagittal injury in asphyxia at term. Eur. J. Paediatr. Neurol..

[27] Martinez-Biarge M., Bregant T., Wusthoff C., Chew A.T., Diez-Sebastian J., Rutherford M.A., Cowan F.M. (2012). White matter and cortical injury in hypoxic-ischemic encephalopathy: Antecedent factors and 2-year outcome. J. Pediatr..

[28] Harteman J.C., Groenendaal F., Toet M.C., Benders M.J., Van Haastert I.C., Nievelstein R.A., Koopman-Esseboom C., De Vries L.S. (2013). Diffusion-weighted imaging changes in cerebral watershed distribution following neonatal encephalopathy are not invariably associated with an adverse outcome. Dev. Med. Child Neurol..

[29] Perez A., Ritter S., Brotschi B., Werner H., Caflisch J., Martin E., Latal B. (2013). Long-term neurodevelopmental outcome with hypoxic-ischemic encephalopathy. J. Pediatr..

[30] Lee B.L., Gano D., Rogers E.E., Xu D., Cox S., Barkovich A.J., Li Y., Ferriero D.M., Glass H.C. (2021). Long-term cognitive outcomes in term newborns with watershed injury caused by neonatal encephalopathy. Pediatr. Res..

[31] Hayman M., van Wezel-Meijler G., van Straaten H., Brilstra E., Groenendaal F., de Vries L.S. (2019). Punctate white-matter lesions in the full-term newborn: Underlying aetiology and outcome. Eur. J. Paediatr. Neurol..

[32] Li A.M., Chau V., Poskitt K.J., Sargent M.A., Lupton B.A., Hill A., Roland E., Miller S.P. (2009). White matter injury in term newborns with neonatal encephalopathy. Pediatr. Res..

[33] Vermeulen R.J., Fetter W.P., Hendrikx L., Van Schie P.E., van der Knaap M.S., Barkhof F. (2003). Diffusion-weighted MRI in severe neonatal hypoxic ischaemia: The white cerebrum. Neuropediatrics.

[34] Annink K.V., Meerts L., van der Aa N.E., Alderliesten T., Nikkels P.G.J., Nijboer C.H.A., Groenendaal F., de Vries L.S., Benders M.J.N.L., Hoebeek F.E. (2021). Cerebellar injury in term neonates with hypoxic-ischemic encephalopathy is underestimated. Pediatr. Res..

[35] Lemmon M.E., Wagner M.W., Bosemani T., Carson K.A., Northington F.J., Huisman T.A., Poretti A. (2017). Diffusion tensor imaging detects occult cerebellar injury in severe neonatal hypoxic-ischemic encephalopathy. Dev. Neurosci..

[36] Radicioni M., Bini V., Chiarini P., Fantauzzi A., Leone F., Scattoni R., Camerini P.G. (2017). Cerebral sinovenous thrombosis in the asphyxiated cooled infants: A prospective observational study. Pediatr. Neurol..

[37] Cowan F., Rutherford M., Groenendaal F., Eken P., Mercuri E., Bydder G.M., Meiners L.C., Dubowitz L.M., de Vries L.S. (2003). Origin and timing of brain lesions in term infants with neonatal encephalopathy. Lancet.

[38] Ramaswamy V., Miller S.P., Barkovich A.J., Partridge J.C., Ferriero D.M. (2004). Perinatal stroke in term infants with neonatal encephalopathy. Neurology.

[39] Adhami F., Liao G., Morozov Y.M., Schloemer A., Schmithorst V.J., Lorenz J.N., Dunn R.S., Vorhees C.V., Wills-Karp M., Degen J.L. (2006). Cerebral ischemia-hypoxia induces intravascular coagulation and autophagy. Am. J. Pathol..

[40] Michoulas A., Basheer S.N., Roland E.H., Poskitt K., Miller S., Hill A. (2011). The role of hypoxia-ischemia in term newborns with arterial stroke. Pediatr. Neurol..

[41] Harbert M.J., Tam E.W.Y., Glass H.C., Bonifacio S.L., Haeusslein L.A., Barkovich A.J., Jeremy R.J., Rogers E.E., Glidden D., Ferriero D.M. (2011). Hypothermia is correlated with seizure absence in perinatal stroke. J. Child Neurol..

[42] Alderliesten T., Nikkels P.G., Benders M.J., de Vries L.S., Groenendaal F. (2012). Antemortem cranial MRI compared with postmortem histopathologic examination of the brain in term infants with neonatal encephalopathy following perinatal asphyxia. Arch. Dis. Child. Fetal Neonatal Ed..

[43] Annink K.V., De Vries L.S., Groenendaal F., Heuvel M.P.V.D., Van Haren N.E.M., Swaab H., Van Handel M., Jongmans M.J., Benders M.J., Van Der Aa N.E. (2019). The long-term effect of perinatal asphyxia on hippocampal volumes. Pediatr. Res..

[44] Molavi M., Vann S.D., de Vries L.S., Groenendaal F., Lequin M. (2019). Signal change in the mammillary bodies after perinatal asphyxia. AJNR Am. J. Neuroradiol..

[45] Lequin M., Steggerda S., Severino M., Tortora D., Parodi A., Ramenghi L.A., Groenendaal F., Meys K.M., Benders M.J., de Vries L.S. (2021). Mammillary body injury in neonatal encephalopathy: A multicentre, retrospective study. Pediatr. Res..

[46] Annink K.V., de Vries L.S., Groenendaal F., Eijsermans R.M.J.C., Mocking M., van Schooneveld M.M.J., Dudink J., van Straaten H.L.M., Benders M.J.N.L., Lequin M. (2021). Mammillary body atrophy and other MRI correlates of school-age outcome following neonatal hypoxic-ischemic encephalopathy. Sci. Rep..

[47] Barkovich A.J., Truwit C.L. (1990). Brain damage from perinatal asphyxia: Correlation of MR findings with gestational age. AJNR Am. J. Neuroradiol..

[48] Shroff M.M., Soares-Fernandes J.P., Whyte H., Raybaud C. (2010). MR imaging for diagnostic evaluation of encephalopathy in the newborn. Radiographics.

[49] Barkovich A.J., Westmark K., Partridge C., Sola A., Ferriero D.M. (1995). Perinatal asphyxia—MR findings in the first 10 days. Am. J. Neuroradiol..

[50] Rutherford M.A., Pennock J.M., Counsell S.J., Mercuri E., Cowan F.M., Dubowitz L.M.S., Edwards A.D. (1998). Abnormal magnetic resonance signal in the internal capsule predicts poor neurodevelopmental outcome in infants with hypoxic-ischemic encephalopathy. Pediatrics.

[51] Sener R.N. (2001). Diffusion MRI: Apparent diffusion coefficient (ADC) values in the normal brain and a classification of brain disorders based on ADC values. Comput. Med. Imaging Graph..

[52] Cowan F.M., Pennock J.M., Hanrahan J.D., Manji K.P., Edwards A.D. (1994). Early detection of cerebral infarction and hypoxic ischemic encephalopathy in neonates using diffusion-weighted magnetic resonance imaging. Neuropediatrics.

[53] Robertson R.L., Ben-Sira L., Barnes P.D., Mulkern R.V., Robson C.D., Maier S.E., Rivkin M.J., Du Plessis A.J. (1999). MR line-scan diffusion-weighted imaging of term neonates with perinatal brain ischemia. AJNR Am. J. Neuroradiol..

[54] Bednarek N., Mathur A., Inder T., Wilkinson J., Neil J., Shimony J. (2012). Impact of therapeutic hypothermia on MRI diffusion changes in neonatal encephalopathy. Neurology.

[55] Haacke E.M., Mittal S., Wu Z., Neelavalli J., Cheng Y.C. (2009). Susceptibility-weighted imaging: Technical aspects and clinical applications, part 1. AJNR Am. J. Neuroradiol..

[56] Bosemani T., Poretti A., Huisman T.A. (2014). Susceptibility-weighted imaging in pediatric neuroimaging. J. Magn. Reson. Imaging.

[57] Kitamura G., Kido D., Wycliffe N., Jacobson J.P., Oyoyo U., Ashwal S. (2011). Hypoxic-ischemic injury: Utility of susceptibility-weighted imaging. Pediatr. Neurol..

[58] Messina S.A., Poretti A., Tekes A., Robertson C., Johnston M.V., Huisman T.A. (2014). Early predictive value of susceptibility weighted imaging (SWI) in pediatric hypoxic-ischemic injury. J. Neuroimaging.

[59] Smith S.M., Jenkinson M., Johansen-Berg H., Rueckert D., Nichols T.E., Mackay C., Watkins K., Ciccarelli O., Cader M.Z., Matthews P.M. (2006). Tract-based spatial statistics: Voxelwise analysis of multi-subject diffusion data. Neuroimage.

[60] Porter E.J., Counsell S.J., Edwards A.D., Allsop J., Azzopardi D. (2010). Tract-based spatial statistics of magnetic resonance images to assess disease and treatment effects in perinatal asphyxial encephalopathy. Pediatr. Res..

[61] Petersen E.T., Zimine I., Ho Y.C., Golay X. (2006). Non-invasive measurement of perfusion: A critical review of arterial spin labelling techniques. Br. J. Radiol..

[62] Kleuskens D.G., Goncalves Costa F., Annink K.V., van den Hoogen A., Alderliesten T., Groenendaal F., Benders M.J., Dudink J. (2021). Pathophysiology of cerebral hyperperfusion in term neonates with hypoxic-ischemic encephalopathy: A systematic review for future research. Front. Pediatr..

[63] Wintermark P., Hansen A., Gregas M.C., Soul J., Labrecque M., Robertson R.L., Warfield S.K. (2011). Brain perfusion in asphyxiated newborns treated with therapeutic hypothermia. AJNR Am. J. Neuroradiol..

[64] Meng L., Wang Q., Li Y., Ma X., Li W., Wang Q. (2021). Diagnostic performance of arterial spin-labeled perfusion imaging and diffusion-weighted imaging in full-term neonatal hypoxic-ischemic encephalopathy. J. Integr. Neurosci..

[65] Proisy M., Corouge I., Legouhy A., Nicolas A., Charon V., Mazille N., Leroux S., Bruneau B., Barillot C., Ferré J.-C. (2019). Changes in brain perfusion in successive arterial spin labeling MRI scans in neonates with hypoxic-ischemic encephalopathy. Neuroimage Clin..

[66] Peden C.J., Cowan F.M., Bryant D.J., Sargentoni J., Cox I.J., Menon D.K., Gadian D.G., Bell J.D., Dubowitz L.M. (1990). Proton MR spectroscopy of the brain in infants. J. Comput. Assist. Tomogr..

[67] Schmitz B., Wang X., Barker P.B., Pilatus U., Bronzlik P., Dadak M., Kahl K.G., Lanfermann H., Ding X.-Q. (2018). Effects of aging on the human brain: A proton and phosphorus MR spectroscopy study at 3T. J. Neuroimaging.

[68] Shibasaki J., Aida N., Morisaki N., Tomiyasu M., Nishi Y., Toyoshima K. (2018). Changes in brain metabolite concentrations after neonatal hypoxic-ischemic encephalopathy. Radiology.

[69] Toft P.B., Leth H., Lou H.C., Pryds O., Henriksen O. (1994). Metabolite concentrations in the developing brain estimated with proton MR spectroscopy. J. Magn. Reson. Imaging.

[70] Roelants-Van Rijn A.M., Van der Grond J., De Vries L.S., Groenendaal F. (2001). Value of H-1-MRS using different echo times in neonates with cerebral hypoxia-ischemia. Pediatr. Res..

[71] Wu T.W., Tamrazi B., Hsu K.H., Ho E., Reitman A.J., Borzage M., Blüml S., Wisnowski J.L. (2018). Cerebral lactate concentration in neonatal hypoxic-ischemic encephalopathy: In relation to time, characteristic of injury, and serum lactate concentration. Front. Neurol..

[72] Shibasaki J., Niwa T., Piedvache A., Tomiyasu M., Morisaki N., Fujii Y., Toyoshima K., Aida N. (2021). Comparison of predictive values of magnetic resonance biomarkers based on scan timing in neonatal encephalopathy following therapeutic hypothermia. J. Pediatr..

[73] Leth H., Toft P.B., Pryds O., Peitersen B., Lou H.C., Henriksen O. (1995). Brain lactate in preterm and growth-retarded neonates. Acta Paediatr..

[74] Roelants-van Rijn A.M., van der Grond J., Stigter R.H., de Vries L.S., Groenendaal F. (2004). Cerebral structure and metabolism and long-term outcome in small-for-gestational-age preterm neonates. Pediatr. Res..

[75] Moorcraft J., Bolas N.M., Ives N.K., Ouwerkerk R., Smyth J., Rajagopalan B., Hope P.L., Radda G.K. (1991). Global and depth resolved phosphorus magnetic resonance spectroscopy to predict outcome after birth asphyxia. Arch. Dis. Child..

[76] Roth S.C., Edwards A.D., Cady E.B., Delpy D.T., Wyatt J.S., Azzopardi D., Baudin J., Townsend J., Stewart A.L., Reynolds E.O.R. (1992). Relation between cerebral oxidative-metabolism following birth asphyxia, and neurodevelopmental outcome and brain growth at one year. Dev. Med. Child Neurol..

[77] Poretti A., Blaser S.I., Lequin M.H., Fatemi A., Meoded A., Northington F.J., Boltshauser E., Huisman T.A. (2012). Neonatal neuroimaging findings in inborn errors of metabolism. J. Magn. Reson. Imaging.

[78] Sener R.N. (2007). Maple syrup urine disease: Diffusion MRI, and proton MR spectroscopy findings. Comput. Med. Imaging Graph..

[79] McAdams R.M., Richards T.L. (2009). Detection of nonketotic hyperglycinemia in a neonate using proton magnetic resonance spectroscopy. Radiol. Case Rep..

[80] Vijayakumar K., Gunny R., Grunewald S., Carr L., Chong K.W., DeVile C., Robinson R., McSweeney N., Prabhakar P. (2011). Clinical neuroimaging features and outcome in molybdenum cofactor deficiency. Pediatr. Neurol..

[81] Durmaz M.S., Ozbakir B. (2018). Molybdenum cofactor deficiency: Neuroimaging findings. Radiol. Case Rep..

[82] Luptakova D., Baciak L., Pluhacek T., Skriba A., Sediva B., Havlicek V., Juranek I. (2018). Membrane depolarization and aberrant lipid distributions in the neonatal rat brain following hypoxic-ischaemic insult. Sci. Rep..

[83] Barkovich A.J., Hajnal B.L., Vigneron D., Sola A., Partridge J.C., Allen F., Ferriero D.M. (1998). Prediction of neuromotor outcome in perinatal asphyxia: Evaluation of MR scoring systems. AJNR Am. J. Neuroradiol..

[84] Shankaran S., Barnes P.D., Hintz S.R., Laptook A.R., Zaterka-Baxter K.M., McDonald S.A., Ehrenkranz R.A., Walsh M.C., Tyson J.E., Donovan E.F. (2012). Brain injury following trial of hypothermia for neonatal hypoxic-ischaemic encephalopathy. Arch. Dis. Child. Fetal Neonatal Ed..

[85] Trivedi S.B., Vesoulis Z., Rao R., Liao S.M., Shimony J.S., McKinstry R.C., Mathur A.M. (2017). A validated clinical MRI injury scoring system in neonatal hypoxic-ischemic encephalopathy. Pediatr. Radiol..

[86] Weeke L.C., Groenendaal F., Mudigonda K., Blennow M., Lequin M.H., Meiners L.C., van Haastert I.C., Benders M.J., Hallberg B., de Vries L.S. (2018). A novel magnetic resonance imaging score predicts neurodevelopmental outcome after perinatal asphyxia and therapeutic hypothermia. J. Pediatr..

[87] Bhroin M.N., Kelly L., Sweetman D., Aslam S., O'Dea M.I., Hurley T., Slevin M., Murphy J., Byrne A.T., Colleran G. (2022). Relationship between MRI scoring systems and neurodevelopmental outcome at two years in infants with neonatal encephalopathy. Pediatr. Neurol..

[88] Barkovich A., Miller S., Bartha A., Newton N., Hamrick S., Mukherjee P., Glenn O., Xu D., Partridge J., Ferriero D. (2006). MR imaging, MR spectroscopy, and diffusion tensor imaging of sequential studies in neonates with encephalopathy. AJNR Am. J. Neuroradiol..

[89] Skranes J.H., Cowan F.M., Stiris T., Fugelseth D., Thoresen M., Server A. (2015). Brain imaging in cooled encephalopathic neonates does not differ between four and 11 days after birth. Acta Paediatr..

[90] Wintermark P., Hansen A., Soul J., Labrecque M., Robertson R.L., Warfield S.K. (2010). Early versus late MRI in asphyxiated newborns treated with hypothermia. Arch. Dis. Child. Fetal Neonatal Ed..

[91] O’Kane A., Vezina G., Chang T., Bendush N., Ridore M., Gai J., Bost J., Glass P., Massaro A.N. (2021). Early versus late brain magnetic resonance imaging after neonatal hypoxic ischemic encephalopathy treated with therapeutic hypothermia. J. Pediatr..

[92] Barkovich A.J., Baranski K., Vigneron D., Partridge J.C., Hallam D.K., Hajnal B.L., Ferriero D.M. (1999). Proton MR spectroscopy for the evaluation of brain injury in asphyxiated, term neonates. AJNR Am J Neuroradiol..

[93] Penrice J., Lorek A., Cady E.B., Amess P.N., Wylezinska M., Cooper C.E., D’Souza P., Brown G.C., Kirkbride V., Edwards A.D. (1997). Proton magnetic resonance spectroscopy of the brain during acute hypoxia-ischemia and delayed cerebral energy failure in the newborn piglet. Pediatr. Res..

[94] Wintermark P., Labrecque M., Warfield S.K., DeHart S., Hansen A. (2010). Can induced hypothermia be assured during brain MRI in neonates with hypoxic-ischemic encephalopathy?. Pediatr. Radiol..

[95] Wu T.W., McLean C., Friedlich P., Grimm J., Bluml S., Seri I. (2014). Maintenance of whole-body therapeutic hypothermia during patient transport and magnetic resonance imaging. Pediatr. Radiol..

[96] Wezel-Meijler G., de Vries L.S. (2014). Cranial ultrasound—Optimizing utility in the NICU. Curr. Pediatr. Rev..

[97] Hall P., Adami H.-O., Trichopoulos D., Pedersen N.L., Lagiou P., Ekbom A., Ingvar M., Lundell M., Granath F. (2004). Effect of low doses of ionising radiation in infancy on cognitive function in adulthood: Swedish population based cohort study. BMJ.

[98] Pearce M.S., Salotti J.A., Little M.P., McHugh K., Lee C., Kim K.P., Howe N.L., Ronckers C.M., Rajaraman P., Craft A.W. (2012). Radiation exposure from CT scans in childhood and subsequent risk of leukaemia and brain tumours: A retrospective cohort study. Lancet.

[99] (2014). Executive summary: Neonatal encephalopathy and neurologic outcome, second edition. Report of the American college of obstetricians and gynecologists’ task force on neonatal encephalopathy. Obstet. Gynecol..

[100] Wisnowski J.L., Wintermark P., Bonifacio S.L., Smyser C.D., Barkovich A.J., Edwards A.D., de Vries L.S., Inder T.E., Chau V. (2021). Neuroimaging in the term newborn with neonatal encephalopathy. Semin. Fetal Neonatal Med..

[101] de Vries L.S., Alderliesten T., Benders M.J., Groenendaal F. (2016). Serial 1- and 2-dimensional cerebral MRI measurements in full-term infants after perinatal asphyxia. Neonatology.

[102] Byrne P., Welch R., Johnson M.A., Darrah J., Piper M. (1990). Serial magnetic resonance imaging in neonatal hypoxic-ischemic encephalopathy. J. Pediatr..

[103] Millet V., Bartoli J.M., Lacroze V., Raybaud C., Unal D., Girard N. (1998). Predictive significance of magnetic resonance imaging at 4 months of adjusted age in infants after a perinatal neurologic insult. Biol. Neonate.

